# Autologous Fat Injection Laryngoplasty for Unilateral Vocal Fold Paralysis

**DOI:** 10.3390/jcm10215034

**Published:** 2021-10-28

**Authors:** Wen-Dien Chang, Sheng-Hwa Chen, Ming-Hsui Tsai, Yung-An Tsou

**Affiliations:** 1 Department of Sport Performance, National Taiwan University of Sport, Taichung 404401, Taiwan; changwendien@ntus.edu.tw; 2 Department of Audiology and Speech-Language Pathology, Asia University, Taichung 41354, Taiwan; shchen@asia.edu.tw (S.-H.C.); minghsui@mail.cmuh.org.tw (M.-H.T.); 3 Department of Otolaryngology-Head and Neck Surgery, China Medical University Hospital, Taichung 40402, Taiwan; 4 School of Medicine, China Medical University, Taichung 40402, Taiwan

**Keywords:** autologous fat injection laryngoplasty, unilateral vocal fold paralysis, acoustic analysis

## Abstract

Background: Unilateral vocal palsy (UVFP) affects the voice and swallowing function and could be treated by various materials to achieve improved mucosal wave and better closure during phonation. Injection laryngoplasty is considered an exemplary method for these patients and could be injected as early as possible. We conducted a systematic review and meta-analysis for the subjective and objective outcomes of autologous fat injection laryngoplasty (AFIL) and assessed the effects for patients with UVFP. Methods: We searched studies from PubMed and EBSCO databases with PRISMA appraisal to search for articles about the effects of AFIL on UVFP. The published articles were reviewed according to our inclusion and exclusion criteria. The short- and long-term outcomes of perceptual, acoustic analysis, and quality of life were also analyzed by meta-analysis. Results: Eleven articles were reviewed, and seven studies were selected for meta-analysis. AFIL improves the perceptual outcome and some voice parameters in short-term and long-term results, i.e., jitter, shimmer, and maximal phonation time (MPT). It also significantly improved the voice handicap index (VHI) in the long term, suggesting an increase in quality of life. Conclusions: AFIL is considered a reliable treatment method for UVFP and could even last for over 12 months.

## 1. Introduction

Quality of life is compromised in speech and swallowing in patients with unilateral vocal fold paralysis (UVFP). Because of recurrent laryngeal nerve neuropathy, inadequate glottic closure is frequently found because of idiopathic, neoplasm, or iatrogenic causes [1]. The surgical treatment includes injection laryngoplasty, laryngeal framework surgery, recurrent laryngeal nerve (RLN) re-innervation, and laryngeal pacing [2]. The voice outcomes are similar between injection laryngoplasty and medialization thyroplasty [3]. Surgery is considered after conservation therapy. However, recent laryngology consensus considers early injection laryngoplasty as a prior treatment strategy for patients with UVFP in various etiologies. Injection laryngoplasty offered a better approximation of vocal folds and improved the recovery of mucosa waves by creating better vocal fold contact. Because of better approximation and recovering vocal mucosa waves, there was a decreased laryngeal framework rate after injection laryngoplasty [4]. Other surgical methods, including RLN re-anastomosis and laryngeal framework surgery, could be applied for poor recovery or compensation after voice therapy, or for patients who need repeated laryngoplasty injection [5]. In addition, laryngeal reinnervation could not recover the movement of paralyzed vocal cords, but maintained the vocal tension and vocal fold resistance during phonation. However, it takes four to six months to get a stable voice outcome. It often needs medialization thyroplasty to better refine voice quality and consider a salvage for UVFP after the failure of injection laryngoplasty [6]. Thus, injection laryngoplasty aims to decrease the glottic gap and offer better glottal closure, shortening the voice handicapped condition and leading to quicker restoration of speech vocal quality.

Injection laryngoplasty is considered a conserved, safe, and less invasive temporary surgery that helps recover paralyzed vocal folds compared to laryngeal framework surgery. This management was first presented by Brunings [7]. After time gone by, various kinds of materials, includeing xenograft (silicon, Teflon, calcium hydroxylapatite, buffy coat), homograft (dermis, facial), autograft (autologous fat), and synthetic materials (collagen, geoforms, hyaluronic acid (HA), dermalogen, teflon, calcium hydroxylapatite) have been used for injection laryngoplasty [8]. Autologous fat injection laryngoplasty (AFIL) was first presented in 1998. It is autologous and has almost no tissue rejection reaction after undergoing injection laryngoplasty [9]. Few studies have compared fat to other materials for sustained voice outcome improvement. However, some laryngologists considered fat injection laryngoplasty a permanent effect because of adipose stem cells contained in the autologous fat during its harvesting [10]. The quality of fat is affected by harvesting technology, characteristic differences of individual fat, and tissue reaction of autologous fat to the surrounding laryngeal tissues. In clinical practice, the injection technique, assessments, and outcomes related to AFIL vary. Therefore, we conducted this systemic review and meta-analysis to survey the durability of sustained results, subjective and objective voice qualities after AFIL.

## 2. Materials and Methods

### 2.1. Search Strategy and Data Sources

Based on the Preferred Reporting Items for Systematic Reviews and Meta-Analyses (PRISMA) statement, the electronic databases of PubMed, EMBASE, and Cochrane Library and were searched primarily based on the combination of the following keywords: “autologous fat injection”, “laryngoplasty”, “unilateral vocal fold paralysis,” “voice quality”, and “prognosis” up to March 2020 and published in English. The searched literature was limited to adults aged 18 to 80 years. The syntaxes were adjusted according to different databases. The reference list of retrieved articles was scanned for all relevant additional articles and reviews by two researchers. The syntaxes for searches in PubMed and EMBASE were as follows: In the PubMed database, the articles search strategy was conducted #1 (unilateral vocal fold paralysis [All Fields] OR autologous fat injection [All Fields]), #2 (vocal fold paralysis [MeSH Term] OR laryngoplasty [MeSH Term]), #3 (autologous fat injection OR voice quality, prognosis [MeSH Terms]), and #4 (#1, #2 AND #3). In EMBASE database, the strategy was used #1 (unilateral vocal fold paralysis exp OR autologous fat injection), #2 (vocal fold paralysis OR laryngoplasty), and #3 (#1 AND #2).

### 2.2. Study Selection Criteria

Articles about the outcomes of AFIL were searched independently by two researchers. Criteria included were articles about UVFP patients diagnosed either by laryngoscope or stroboscopy and treated only by AFIL, and had assessment outcomes before and after AFIL and at follow-up. Exclusion criteria were that the article is a review study or case report and was not published in English. The suitable data of outcomes in the included articles were reviewed and extracted for meta-analysis.

### 2.3. Data Extraction and Quality Assessment

Data regarding the first author, article publication year, study designs, and medical data, including anesthesia, fat harvesting site, injection preparation, preparation volume of fat, injection approach, and injection guidance, were extracted and collected. Data were extracted by two researchers independently using a standardized format. The outcome assessments for AFIL were classified as perceptual, acoustic analysis, and quality of life. Subject data in the cases of UVFP treated by AFIL, i.e., reviews assessment times and outcomes, were extracted directly from the data provided in the included articles. As the original publication provided the median value of VHI-10, MPT, GRBAS, jitter, shimmer, noise harmonic ratio (NHR), and F0 (fundamental frequency), one researcher was directly contacted to get more information for meta-analysis. The publication bias was assessed by Rosenthal’s fail-safe number. The bias risk of the included study used the Cochrane risk of bias tool to assess article quality. The level of “low bias”, “unclear”, or “high bias” were scored for each element to evaluate methodological quality by two researchers.

### 2.4. Statistical Analysis

MedCalc software (MedCalc, Mariakerke, Belgium) was used to perform statistical analysis. In meta-analysis, the effect sizes were used to analyze the values of the outcomes before and after AFIL as the main measure of association. The treatment effects on the assessment variables were analyzed at different time points after AFIL, which were divided into short-term (≤6 months), medium-term (>six months and <12 months), and long-term (≥12 months). The pooling of the extracted data from the articles is calculated in fixed or random-effects models. The estimates of standardized mean difference (SMD) with 95% confidence intervals (CIs) were represented. The Q statistic and I2 statistics were used for heterogeneity analysis. All analysis with *p* < 0.05 was considered statistically significant.

## 3. Results

### 3.1. Data Search Results

**Figure 1:**
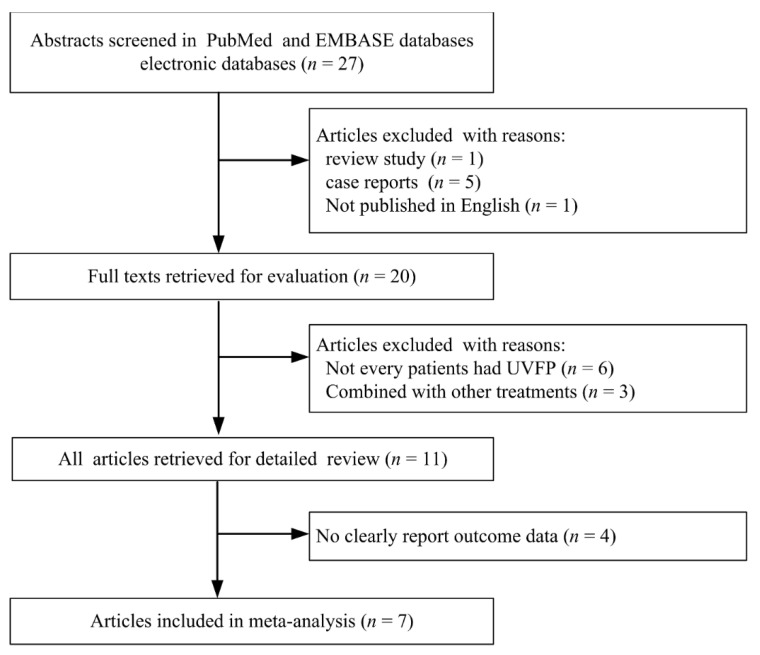
Flow diagram of this study.

**Figure 2:**
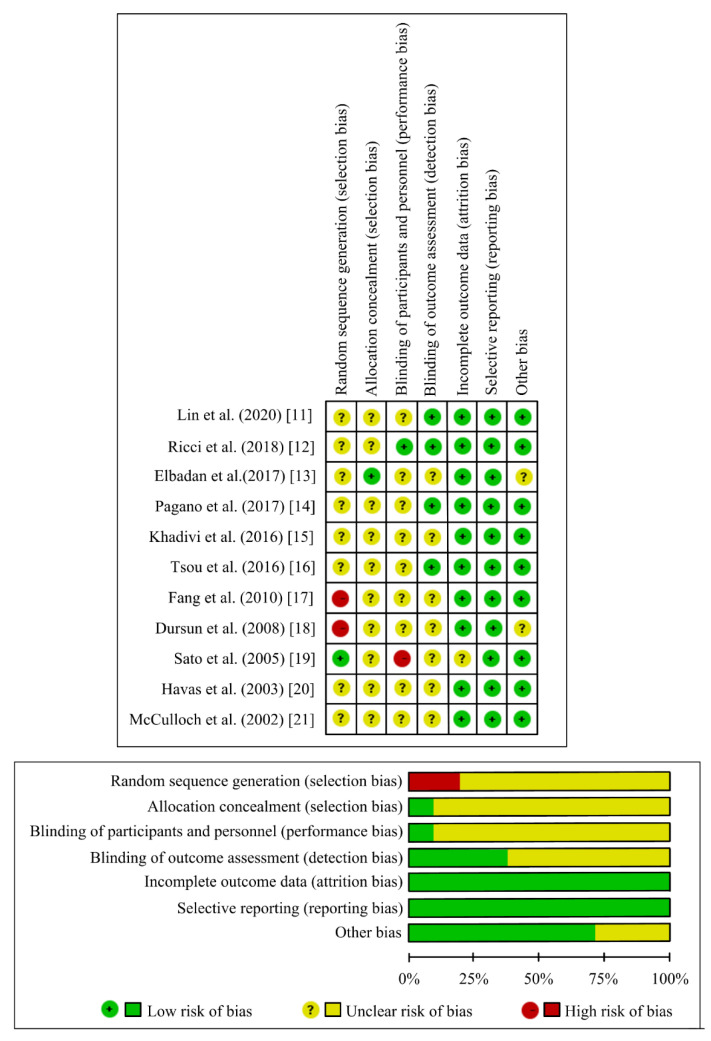
Summary of the risk of bias in 11 included articles.

Twenty-seven articles about autologous fat injection laryngoplasty on UVFP were searched from two electronic databases. Following reviews with the two investigators, 20 articles were included as the study selection criteria (Figure 1). One review study, five case reports, and one non-English article were excluded. The full texts of 20 included articles were reviewed again. However, nine of them were excluded because not all patients had UVFP and fat injection laryngoplasty combined with other treatments. Eleven clinical studies about autologous fat injection laryngoplasty for UVFP were reviewed [11,12,13,14,15,16,17,18,19,20,21], and the risk of bias for all articles is summarized in Figure 2. Finally, seven studies were suitable for meta-analysis.

### 3.2. Study Characteristics

All included articles were published between 2002 and 2020 [11,12,13,14,15,16,17,18,19,20,21], and the study characteristics were summarized in Table 1. For the 11 included articles, there were three retrospective studies [13,14,21], and eight prospective studies [11,12,15,16,17,18,19,20] in Table 1. A total of UVFP patients (*n* = 337) received autologous fat injection laryngoplasty and were assessed for surgical outcomes. The surgeries were undertaken under general [11,13,14,15,18] or local anesthesia [12,17,20], and the fat was harvested from the abdomen [11,12,13,14,15,16,17,19,20,21] or thigh area [14]. The harvested fat was managed by saline washing [11,13,16,17,19] or concentration [12,14,15]. Four articles had no specific record [18,19,20,21], and seven articles used two approaches [11,12,13,14,15,16,17], including trans-oral in two (28.57%) studies [11,16] and trans-cutaneous in five (71.42%) studies [12,13,14,15,17]. In summary, the injection approach injected the 0.3–5 mL fat transcutaneously or transorally under the device guidance [11,12,13,14,15,16,17,19]. For the 11 articles that reported using injection approaches, AFIL was performed by trans-oral approach in two articles, (18.18%) [11,16] by trains-cutaneous approach in five articles (45.45%) [12,13,14,15,17], and the AFIL approach was not reported in four articles (36.36%) [18,19,20,21].

### 3.3. Study Outcomes

The assessments for perceptual, acoustic analysis and quality of life were evaluated pre- and post-operation (Table 2). For 11 articles, GRBAS were used to assess the perceptual outcome in seven articles (63.63%) [11,12,13,14,16,18,21], and four articles (36.36%) did not report [15,17,19,20]. In GRBAS, five items, including grade, roughness, breathiness, asthenia, and strain were consisted and followed scale as 0 (normal), 1 (mild), 2 (moderate), and 3 (severe). The voice parameters in the acoustic analysis were assessed in 10 of 11 articles (90.90%) [11,12,13,14,15,16,17,18,19,20], and one article (9.10%) did not report [21]. Acoustic analysis was conducted in the voice laboratory [11,12,13,14,15,16,17,18,19,20], and the voice parameters were objectively assessed for voice quality. For voice parameters, the F0, jitter, shimmer, MPT, and NHR were reported. Three articles (27.27%) used VHI for quality of life assessment [1,2,4], but eight articles (72.72%) did not report [13,15,16,17,18,19,20,21]. The assessment time was undertaken pre-and post-operation as early as one week to 12 months. The length of assessment times was divided into short-term (one to six months) and long-term (≥12 months) for the meta-analysis. Eight of 11 articles (72.72%) reported that GRBAS and voice parameters were improved after AFIL [11,12,13,14,15,16,17,18].

### 3.4. Outcomes of Meta-Analysis

After reviewing the articles, seven articles had sufficient outcome data for meta-analysis [11,13,14,15,16,17,18], but four articles were excluded because they did not report outcome data [12,19,20,21]. In the follow-up, the perceptual, acoustic analysis, and quality of life data were analyzed and compared in short-term (≤6 months) and long-term (≥12 months) results for the subgroup analysis. Because of insufficient outcome data in the 6~12 months period, the medium-term result is difficult to analyze. The result of Rosenthal’s fail-safe number reported that the tolerance level of 35 was not higher than the fail-safe number of 133 in the seven articles. So, the publication bias did not influence the meta-analysis.

**Figure 3:**
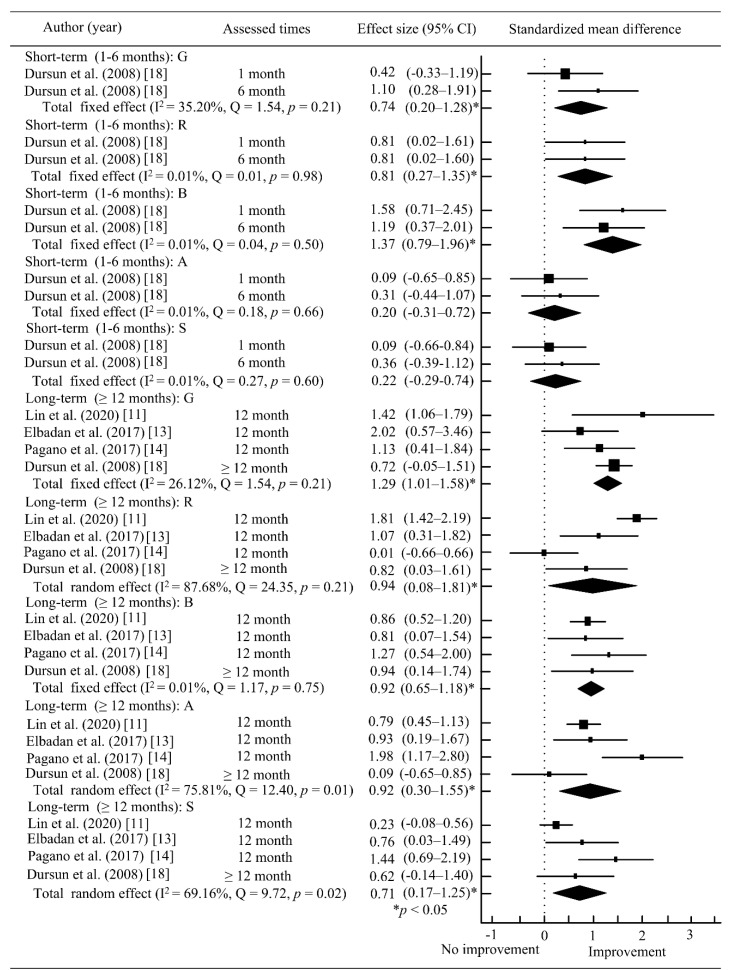
Meta-analysis of GRBAS after AFIL. * *p* < 0.05; ■, SMD; ◆ total SMD.

For the perceptual outcome grading GRBAS (Figure 3), the results of meta-analyses revealed that pooled SMD were statistically significant in G (total SMD = 0.74; 95% CI = 0.20~1.28; *p* < 0.05), R (total SMD = 0.74; 95% CI = 0.20~1.28; *p* < 0.05), and B (total SMD = 0.74; 95% CI = 0.20~1.28; *p* < 0.05) at ≤6 month. The statistically significant in G (total SMD = 1.29; 95% CI = 1.01~1.58; *p* < 0.05), R (total SMD = 0.94; 95% CI = 0.08~1.81; *p* < 0.05), B (total SMD = 0.92; 95% CI = 0.65~1.18; *p* < 0.05), A (total SMD = 0.92; 95% CI = 0.30~1.55; *p* < 0.05), and S (total SMD = 0.71; 95% CI = 0.17~1.25; *p* < 0.05) were also noted at ≥12 months. The between-study heterogeneity ranged from I2 = 0.01% to I2 = 87.68% in the subgroup analysis. The short-term and long-term results suggested that AFIL have the improvement in the perceptual outcome.

**Figure 4:**
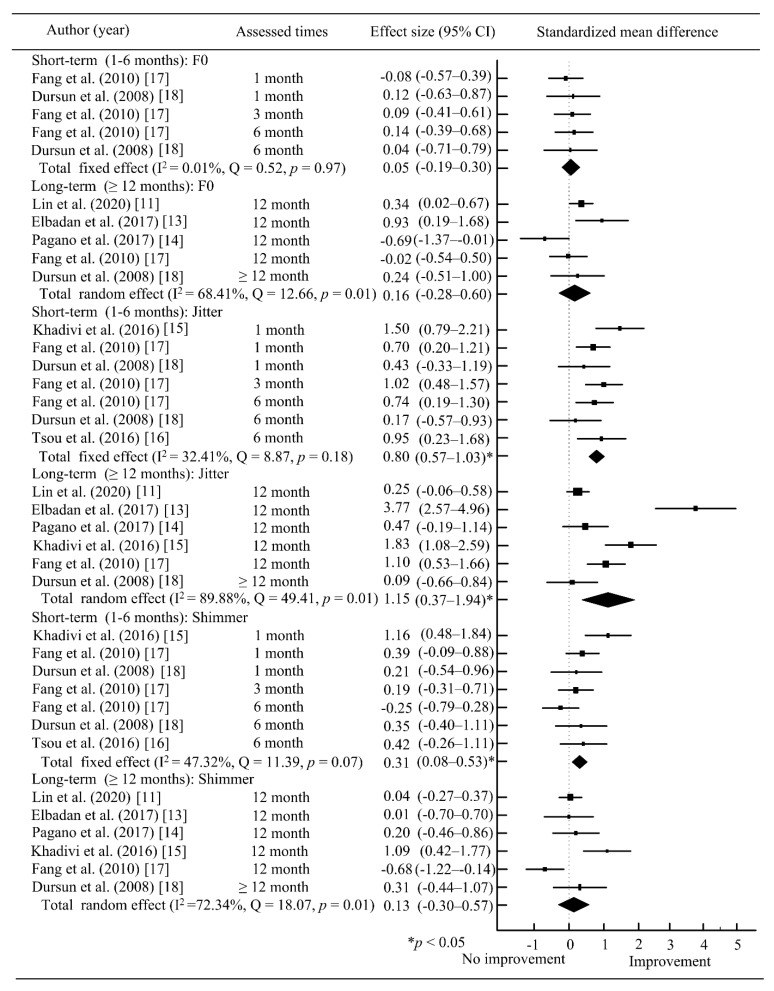
Meta-analysis of voice parameters (F0, jitter, and shimmer) after AFIL. * *p* < 0.05; ■, SMD; ◆ total SMD.

**Figure 5:**
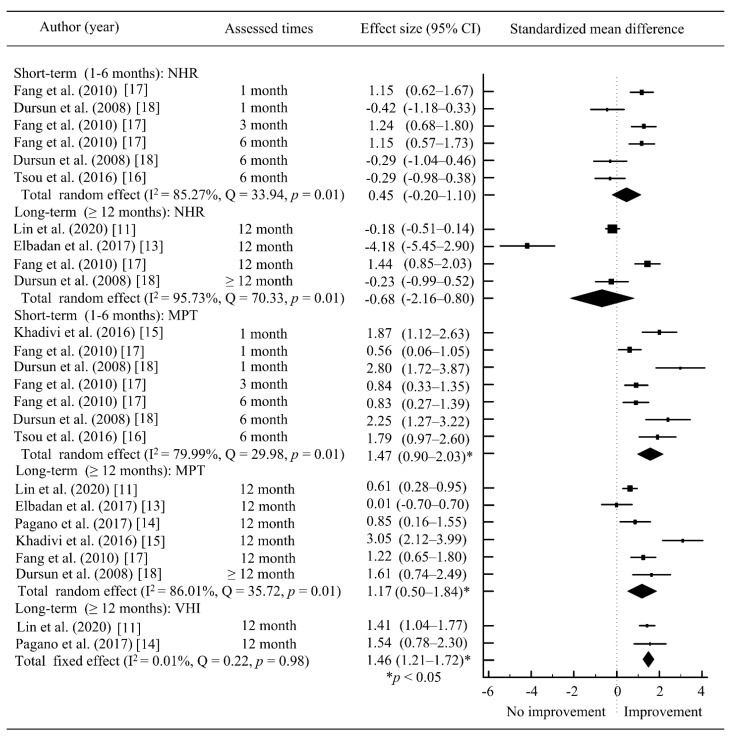
Meta-analysis of voice parameters (NHR and MPT) and quality of life after AFIL. * *p* < 0.05; ■, SMD; ◆ total SMD.

For the outcome in acoustic analysis, the voice parameters (F0, jitter, shimmer, MPT and NHR) were analyzed in Figure 4 and Figure 5. The results of meta-analyses revealed that there were statistically significant in jitter (total SMD = 0.80; 95% CI = 0.57~1.03; *p* < 0.05), shimmer (total SMD = 0.31; 95% CI = 0.08~0.53; *p* < 0.05) and MPT (total SMD = 1.47; 95% CI = 0.90~2.03; *p* < 0.05) at ≤six months. At ≥12 months, pooled SMD were statistically significant in Jitter (total SMD = 1.15; 95% CI = 0.37~1.94; *p* < 0.05), and MPT (total SMD = 1.17; 95% CI = 0.50~1.84; *p* < 0.05). The between-study heterogeneity ranged from I2 = 0.01% to I2 = 95.73% in the subgroup analysis. The short-term and long-term results showed that AFIL had improvement in some voice parameters, i.e., jitter, shimmer and MPT.

The VHI was used in only two articles [11,14]. The result of meta-analyses revealed that there was statistically significant in VHI (total SMD = 1.46; 95% CI = 1.21~1.72; *p* < 0.05) in the long-term period (Figure 5) suggesting an improvement in quality of life. However, there were no short-term data available for VHI.

## 4. Discussion

As far as we know, our study is the first systematic review and meta-analysis of the difference in perceptual, acoustic analysis, and quality of life of UVPF in short-term (≤6 months), medium-term (6~12 months), and long-term (≥12 months) after AFIL. The subjective perceptual and quality of life signified by VHI, GRBAS improved significantly in short-term [12,14,18], medium-term [18], and long-term [11,12,15,18] results after AFIL. The objective voice outcomes by MPT, jitter, shimmer, NHR are also significantly improved in short-term [14,18], medium-term [16,18], and long-term [11,13,15,17,18] results after AFIL. Our meta-analysis result revealed that the AFIL could have a long-term effect in improving the voice outcome in jitter but not shimmer. But we could not draw out a conclusion why jitter matters but shimmer does not. We found that the AFIL could only stabilize the mucosa wave in the long-term outcome; however, the perfect approximation might not be sustained after fat reabsorption; therefore, long-term shimmer outcome benefit could not be gained.

To sum up, we found AFIL improved (VHI and GRBAS) and objective voice outcomes (MPT and jitter) for at least 12 months. However, there were no changes in F0 and NHR by AFIL. Thus we conclude that the AFIL is not changing the vibration rate of bilateral vocal folds, there is no obvious noise reduction component measured by MDVP, and the noise harmonic ratio is not significantly changed after AFIL. However, we consider it is a good treatment choice for patients with UVFP.

The etiology of UVPF is complex and is of inflammation, neoplastic, traumatic, idiopathic, iatrogenic, and neurogenic cause [22]. The recent prior etiologies of UVFP were post-thyroid surgery, idiopathic, and thoracic surgery [23]. There were often mixed etiologies causing the vocal gap and decreased mucosa wave during phonation. Not only does vocal quality affect the patient’s communication function, but it also affects the swallowing function and causing a reduced quality of life [24]. Injection laryngoplasty is considered an exemplary method for these patients, and it could be injected as early as possible [25]. However, many materials could be injected into the vocal area to decrease the vocal gap or slit during phonation to increase the mucosa wave [26]. However, the voice quality and sustainable effect are considered by AFIL. Regardless, the vocal quality and the impact of vocal function and durability of fat are not clear. The sustained voice outcome could be reached up to 12 months [11,12,13,15,17,18] but might decrease after that time [17,21].

The history of injection laryngoplasty was first presented by Dr. Brunings in 1911, more than a century ago [27,28]. Multiple kinds of materials could be injected into the vocal fold thyroarytenoid muscle area presented since 1911. Short-term temporal material for injection laryngoplasty includes bovine gelatin collagen-based products (i.e., Cymetra, Zyplast, Gelfoam, Surgifoam, and Cosmoplast/Cosmoderm) [29], hyaluronic acid (Restylane, and Hylaform), and carboxymethylcellulose (Radiesse Voice Gel) [30]. Ricci et al. indicated that AFIL had no complication during the injection procedure because the material was autologous fat, which caused less inflammation [12]. The AFIL is safe and with good efficacy for UVPF. The materials that had a longer duration with permanent (long-lasting) effects in the body include autologous fat, calcium hydroxylapatite (Radiesse), ArteSense, and particulate silicone [31]. Autologous fat is safe and widely accepted with fewer adverse effects such as umbilical herniation [32]. There were few complications after injection laryngoplasty by collagen, hyaluronic acid, and calcium hydroxylapatite, micronized AlloDerm including infection, laryngeal abscess formation [33,34,35,36], and acute dyspnea by polydimethylsiloxane (PDMS) [37]. Therefore, autologous fat injection laryngoplasty was still considered a proper long-lasting treatment with a fewer complication for patients with UVFP. Because of reports of 50% (45% failure rate after four years) reabsorption after fat injection laryngoplasty after longer run [21]. Therefore, AFIL is preferred over injection, but sometimes contributes to persistent vocal strain and poor voice quality in the initial two to three weeks after AFIL.

In harvesting fat, preventing long-term air exposure is warranted, and better to remove the emissary fat after waiting for 10 min for precipitation after configuration to separate the plasma and liquid oil before injection laryngoplasty [12,13,14,15]. The configuration speed should not be so high, and it is suggested to not exceed over 3000 mph in order to prevent injury to fat cells [14,15]. The centrifuged autologous fat could contain stem cells to increase new adipocytes [12], which may cause long-term effects on perceptual, acoustic analysis, and quality of life in UVPF patients. Sometimes, insulin saturation is applied to autologous fat to increase the survival rate of fat because of the simulation of insulin growth factor in the fat cells [16]. There were also combined materials to mix with fat to improve the survival of fat like PRP that is helpful to the decreased absorption rate of fat. The adipose stem cells could be harvested during harvesting fat; however, the percentage of adipose stem cell (ASC) is not predictable. The younger patients might have a higher concentration of ASC than older patients. Future studies of bone marrow harvesting mesenchymal stem cells or using the growth factors mixtures with fat are warranted.

In our review, studies included suggested RLN, not SLN-related UVFP, and excluded the glottic insufficiency because of the vocal sulcus, vocal atrophy, presbylarynx, or vocal scarring. Thus, this study might enroll UVFP with larger glottal gap but less vocal scarring condition. However, the width of glottal gap is not clearly mentioned in each of the studies. Furthermore, the timing of injection laryngoplasty is different in each study, ranging from one month to 12 months after finding vocal palsy. In the current literature review, the possible spontaneous recovery rate and the complex etiologies of UVPF are also different. However, most of them are iatrogenic. The detailed harvesting methods are different in each study and reviewed by Truzzi et al. [10]. The injection methods are also different for each study; transcutaneous injection and transoral injection, both by suspension laryngoscopy, are the main methods for autologous fat injection laryngoplasty. Compared to transoral and transcutaneous injection laryngoplasty, we could not conclude the voice outcomes according to this study. In addition, the voice outcome quality is based on the quality of fat. However, we could not exactly know the quality of fat harvested in all included studies. Therefore, no conclusive decision could be made between transcutaneous or transoral injection laryngoplasty. In our experience, the amount of fat could be lessened by transcutaneous injection than transoral injection. Thus, the thinner patients could consider transcutaneous injection if the hard harvesting fat condition is met. In addition, most of the injection is under general anesthesia [11,13,14,15,16]. The equipment of injection laryngoplasty includes a No. 18 [11,15,16,19,38], No, 19 [14,20] and No. 22 [12,18] brunings syringe, or even No.18 spinal needle [13]. In patients with UVFP, the need of AFIL is from 0.5 to 5 mL [11,12,13,14,15,16,17,19,20]. AFIL still could be the selected treatment prior to further laryngeal framework surgery. Patients who need repeated AFIL considered a further treatment for laryngeal framework surgery as isshiki type I medialization thyroplasty [39].

AFIL could be a permanent procedure because of harvesting viable adipose stem cells. There were still laryngologists believing that AFIL may be a permanent procedure for UVFP because of higher ASC harvested [40]. That is also a possible explanation as to why AFIL markedly decreased the need for laryngeal framework surgery [41]. In addition, autologous fat material is considered the ideal material. The ideal material is considered to meet the criteria of not causing tissue reactions such as tissue rejection or tissue inflammation. The sustained function to fill the tissue defects. Easy to harvest with reliable to use. In the literature review, the AFIL was widely accepted and the voice outcome is good [11,13,14,15,16,17,18]. The results of our reviewed articles supported that AFIL is a suitable phonosurgical treatment for UVFP. Our meta-analysis results revealed that MPT and jitter were significantly improved in short- and long-term effects after AFIL. The improvement in shimmer was only noted in the short-term result. However, no significant differences in F0 and NHR were foundin short- and long-term results. Elbadan et al. thought that AFIL could reduce the glottal gap size, reducing the flow rate and subglottic pressure [13]. Jitter is presented as the measurable frequency perturbation and an important parameter to assess the improvement in voice quality [11]. Shimmer, F0, and NHR also could reflect the vocal abnormalities and are the indicators of voice quality improvement [11]. This review answers which improvement is gained in subjective and objective voice quality after AFIL and the average duration of the effective outcomes for patients with UVFP. We conclude that AFIL helps with subjective and objective voice quality in short and long-term follow-up with no F0 and NHR changes.

In our review, patient selection suggested RLN, not SLN, and excluded a small gap. Thus, the treatment outcomes measured for UVFP patients are considerable in this review. However, most of the included studies follow the patients from three months to 12 months. Therefore, we could only have divided the outcomes by the short term, defined as six months or within after AFIL, and the long time by 12 months or longer. A longer follow-up study is required. In addition, there are UVFP etiology concerns in our collected studies (different etiologies and different disease durations).

## 5. Conclusions

This study suggests that AFIL significantly improved subjective voice outcome measures by VHI and GRBAS and resulted in prolonged MPT and better jitter in the short and long term. However, there was only improved shimmer after surgery and for six months after, as the effect was not sustained for 12 months. The NHR was not improved by AFIL. There were a few complications and this could be widely considered in patients with UVFP.

## Figures and Tables

**Table 1:** Summary of the characteristics in 11 included articles.

Author (Years)	Study Designs	Patients (*n*)	Anesthesia	Fat Harvesting Site	Injection Preparation	Preparation Volume	Injection Approach	Injection Guidance
Lin et al. (2020) [11]	Prospective study	73	General	Abdomen	Saline washing	2 mL	Trans-oral	Rigid suspension laryngoscope
Ricci et al. (2018) [12]	Prospective study	22	Local	Abdomen	Concentration (3000 rpm for 3 min)	3 mL	Trans-cutaneous	Flexible endoscope
Elbadan et al. (2017) [13]	Retrospective study	16	General	Abdomen	Saline washing and survive use of insulin (100 units)	5 mL	Trans-cutaneous	Laryngoscope
Pagano et al. (2017) [14]	Retrospective study	18	General	Abdomen or thigh	Concentration (3 min)	1 mL	Trans-cutaneous	Surgical microscope
Khadivi et al. (2016) [15]	Prospective study	20	General	Abdomen	Concentration (2000 rpm for 4 min)	2 mL	Trans-cutaneous	Rigid suspension laryngoscope
Tsou et al. (2016) [16]	Prospective study	17	General	Abdomen	Saline washing and survive use of insulin (10 mL)	1.5–2 mL	Trans-oral	Rigid suspension laryngoscope
Fang et al. (2010) [17]	Prospective study	33	Local	Abdomen	Saline washing	0.5–2 mL	Trans-cutaneous	Rigid suspension laryngoscope
Dursun et al. (2008) [18]	Prospective study	30	NA	NA	NA	NA	NA	NA
Sato et al. (2005) [19]	Prospective study	13	NA	Abdomen	Saline washing and antibiotics	2 mL	NA	NA
Havas et al. (2003) [20]	Prospective study	45	Local	Abdomen	NA	0.3–0.9 mL	NA	NA
McCulloch et al. (2002) [21]	Retrospective study	50	NA	Abdomen	NA	NA	NA	NA

**Table 2:** Summary of the assessment, assessment time and outcomes in 11 included articles.

Author (Years)	Assessments			Assessment Times	Outcomes
	Perceptual	Acoustic Analysis	Quality of Life		
Lin et al. (2020) [11]	GRBAS	F0, Jitter, Shimmer, NHR, VTI, SPI	VHI-10	Pre- and post-operation 12 month	Significant improved in GRBAS * and some voice parameters * at 12 month
Ricci et al. (2018) [12]	GRBAS	MPT	VHI-10	Pre- and post-operation 1 week and 6 month	Significant improved in GRBAS * MPT and VHI-10 * at 1 week and 12 month
Elbadan et al. (2017) [13]	GRBAS	F0, Jitter, Shimmer, NHR, MPT, MFR, Psub	nil	Pre- and post-operation 12 month	Significant improved in GRBAS * and voice parameters * at 12 month
Pagano et al. (2017) [14]	GRBAS	F0, MPT, Jitter, Shimmer, MFR, lowest and highest intensity, ADSI	VHI	Pre-, post-operation immediately and 12 month	Significant improved in GRBAS *, some voice parameters * and VHI after operation
Khadivi et al. (2016) [15]	nil	MPT, Jitter, Shimmer	nil	Pre-, post-operation 1, and ≥12 months	Significant improved in voice parameters * post-operation ≥ 12 month
Tsou et al. (2016) [16]	GRBAS	F0, Jitter, Shimmer, NHR, MPT	nil	Pre- and post-operation 6 month	Significant improved in some voice parameters * at 6 month
Fang et al. (2010) [17]	nil	F0, Jitter, Shimmer, NHR, MPT, SZ ratio	nil	Pre-, post-operation 1, 3, 6, and 12 months	Significant improved in voice parameters * at 12 month
Dursun et al. (2008) [18]	GRBAS	F0, Jitter, Shimmer, NHR, MPT	nil	Pre-, post-operation 1, 6, and ≥12 months	Significant improved in GRBAS * and voice parameters * after 1, 6, and ≥12 months
Sato et al. (2005) [19]	nil	MPT, MFR	nil	Post-operation ≥ 6 month	nil
Havas et al. (2003) [20]	nil	MPT, Sydney Voice Clinic voice dysfunction rating scale	nil	2 month–8 years	nil
McCulloch et al. (2002) [21]	GRBAS	nil	nil	Pre-, post-operation ≥ 6 month	nil

MPT: maximal phonation time; NHR: noise harmonic ratio; SPI: soft phonation index; VHI: voice handicap index. * *p* < 0.05.

## Data Availability

Data is contained within the article.

## Abbreviations

UVFP: unilateral vocal palsy; AFIL: autologous fat injection laryngoplasty; MPT: maximal phonation time; VHI: voice handicap index; RLN: recurrent laryngeal nerve; HA: hyaluronic acid; PRISMA: Preferred Reporting Items for Systematic Reviews and Meta-Analyses; NHR: noise harmonic ratio; SMD: standardized mean difference; ASC: adipose stem cell.
